# Morphological, immunohistochemical, and biochemical study on the ameliorative effect of gallic acid against bisphenol A-induced nephrotoxicity in male albino rats

**DOI:** 10.1038/s41598-023-28860-1

**Published:** 2023-01-31

**Authors:** Shaimaa M. M. Saleh, A. Bakr Mahmoud, M. Bassam Al-Salahy, Fatma Ahmed Mohamed Moustafa

**Affiliations:** Department of Zoology and Entomology, Faculty of Science, Assiut University, Asyût, 71516 Egypt

**Keywords:** Biochemistry, Biological techniques, Cell biology, Structural biology, Zoology

## Abstract

This study aimed to determine the effect of gallic acid (GA) on ameliorating bisphenol A (BPA) nephrotoxicity in male rat kidneys. Forty rats were assigned randomly into two groups: control (ten animals) and BPA (40 mg/kg bwt) (thirty animals), the second group was divided into three subgroups: BPA alone, BPA + G50 (50 mg/kg bwt), and BPA + G200 (200 mg/kg bwt). The biochemical analysis included measurements of the contents of nitric oxide, lipid peroxidation, reactive oxygen species, and cytokines (interleukin-1α and interleukin-6) in the kidney. The antioxidant enzymes catalase and superoxide dismutase were also measured in the kidney. Kidney function was assessed by determining uric acid, urea, and creatinine levels. The morphological investigations included hematoxylin and eosin staining for assessing the general histology and determining the glomerular and corpuscular areas, the tubular cell degeneration mean area, and the mean leukocyte infiltration area. Also, collagen fiber intensity and polysaccharide content were analyzed. Furthermore, immunohistochemical, morphometric, and ultrastructural studies were carried out. The results revealed morphological, immunohistochemical, and biochemical alterations in the kidney. Most of these changes showed a satisfactory improvement of kidney damage when BPA-administered rats were treated with GA at both doses. In conclusion, GA exhibited a strong protective effect against BPA-induced nephrotoxicity.

## Introduction

Bisphenol A [BPA; 2, 2-bis (4-hydroxyphenyl) propane] is a precursor of polycarbonates and is widely used in plastic containers. It is also considered an endocrine disrupting chemical. BPA can be found in reusable water bottles, polycarbonate baby bottles, the inner coating of metal food cans, and many other products^1^. Under some conditions, such as high temperatures and acidic or basic conditions, BPA can be released into the air, water, and food from these polycarbonate products. Excessive human exposure to BPA is unavoidable. Inhalation, ingestion, or absorption of BPA has resulted in its blood concentration levels ranging from 10 to 100 NM^2^.

BPA is a xenoestrogen, a substance that exhibits estrogen-like properties^3^_**.**_ There have been many conflicting reports on BPA due to its ability to induce oxidative stress. These reports have described the pro-oxidant/antioxidant behaviour of BPA^4^, antioxidant depletion^5^, mitochondrial dysfunction^6^, and apoptosis^7^. The induction of reactive oxygen species (ROS) by BPA may be carcinogenic and toxic^8^. BPA can also influence cytokine expression via an oestrogen receptor/(ER/)-dependent mechanism, as evidenced by evidence of ER/ antagonist-reversed cytokine expression^9^.

BPA is nephrotoxic, a characteristic related to the accumulation of its toxic metabolites and the inability of the kidney to eliminate these metabolites^10^. It increases lipid peroxidation (LPO) in the kidney, resulting in kidney damage and dysfunction^11^. A study confirmed that BPA induces oxidative stress and enhances the cytokine and chemokine gene expression associated with the immune response. BPA increases tumor necrosis factor- (TNF-) and interleukin-6 (IL-6) production in primary human macrophages^12^. Asahi et al*.*^13^ also reported that BPA induced cell apoptosis in hepatocytes in vitro.

Natural supplements are currently used to treat many diseases for two reasons: they improve drug efficacy and/or minimise their harmful side effects^14^. Similarly, numerous scientific studies have revealed that polyphenol compounds have a variety of pharmacological functions related to the antioxidant system^15^. Gallic acid (GA) and its derivatives belong to a large group of plant polyphenols and are present in nearly every part of the plant. It exists in legumes, vegetables, fruits, and beverages^16^ and is considered a potent free radical scavenger^17^. It is widely used in traditional medicines due to its antiallergic, antimutagenic, anti-inflammatory, antioxidant, and anticancer activities^18^. This study aimed to determine the effect of GA on ameliorating BPA nephrotoxicity in the kidneys of male rats.

## Results

### Biochemical analysis

#### Kidney function

**Figure 1:**
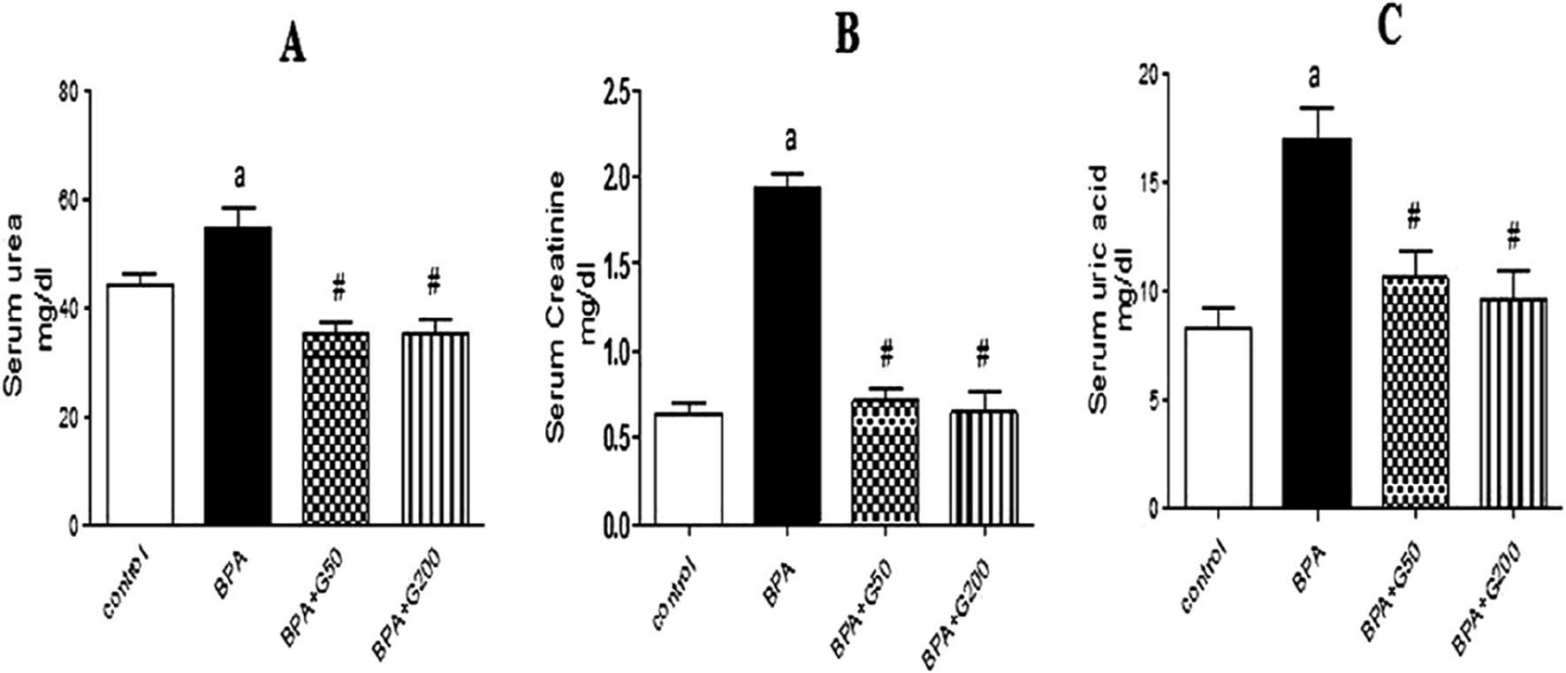
Ameliorative effects of GA (50 mg and 200 mg/kg bwt) on kidney functions, serum urea (**A**), creatinine (**B**), and uric acid (**C**), in rats treated with BPA. Columns with different superscripts are significantly different at *p* < 0.05 (^a^versus control and ^#^versus BPA).

Compared with the control group, there was a significant increase in the levels of urea, creatinine, and uric acid in the BPA group (*p* < 0.05, *p* < 0.001, and *p* < 0.01, respectively). These levels were significantly decreased in the BPA + G50 and BPA + G200 groups compared with the BPA group (*p* < 0.001 and *p* < 0.01, respectively) (Fig. 1a–c).

#### Kidney ROS and NO levels

**Figure 2:**
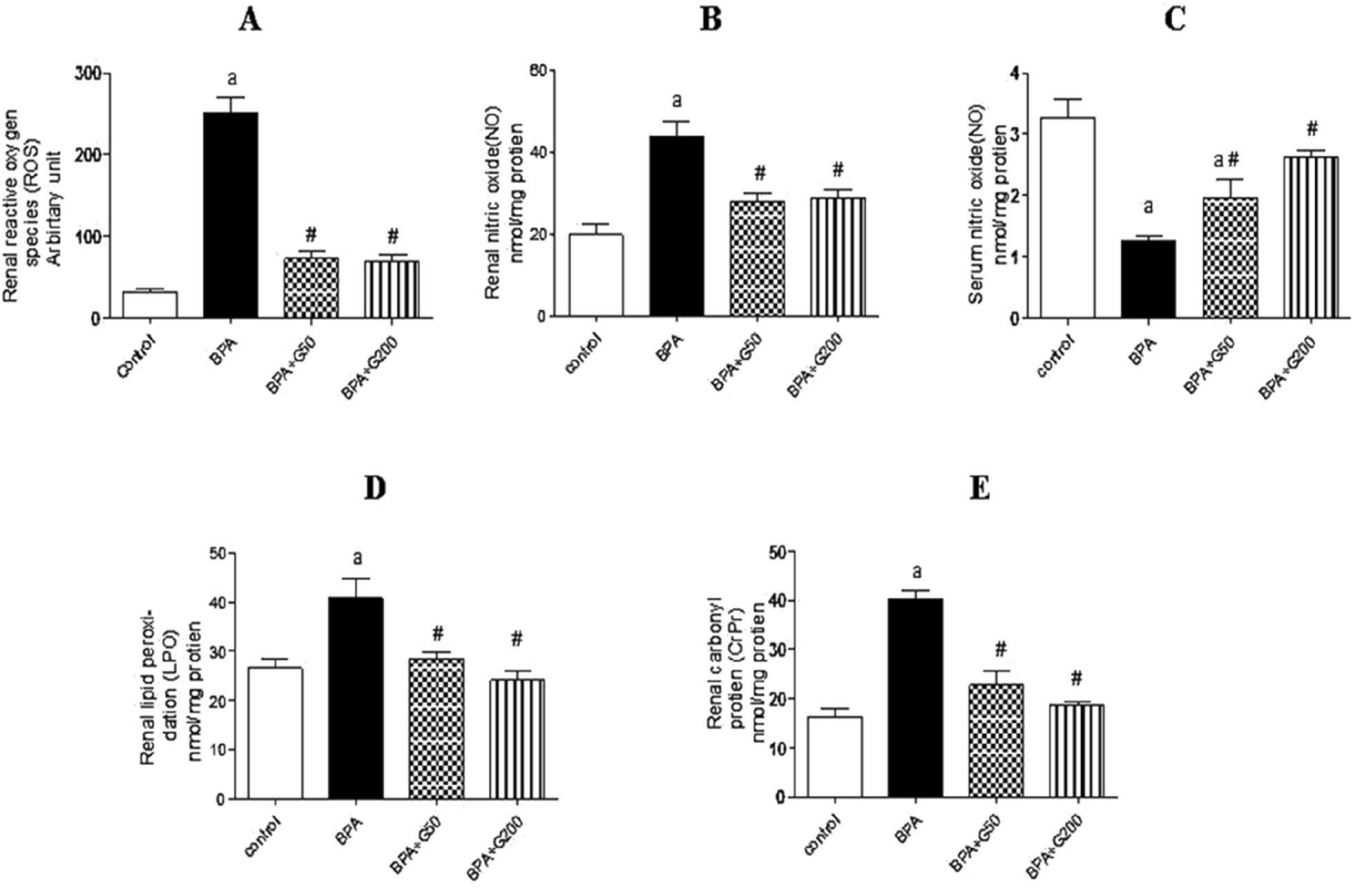
Ameliorative effects of GA (50 mg and 200 mg/kg bwt) on some free radicals. Renal reactive oxygen species (ROS) (**A**), Renal nitric oxide (NO) (**B**), serum nitric oxide (NO) **(C)**, renal lipid peroxidation (LPO) **(D),** and renal carbonyl protein (CrPr) **(E)** levels in the kidneys in rats-treated with BPA. Columns with different superscripts are significantly different at *p* < 0.05 (^a^vs. control and ^#^vs. BPA).

A significant increase in rROS and NO levels was observed in the BPA group compared with the control group (*p* < 0.001). These elevations were significantly decreased in the BPA + G50 and BPA + G200 groups compared with the BPA group (*p* < 0.001 for both) (Fig. 2A,B).

#### Serum NO levels (sNO)

There was a significant decrease in sNO levels in the BPA group compared with the control group (*p* < 0.001). The BPA + G50 group showed relatively significant improvement compared with the BPA group (*p* < 0.05). A normalized value was found for the BPA + G200 group compared with the control and BPA groups (*p* < 0.01 for both) (Fig. 2C).

#### Kidney LPO as malondialdehyde (MDA)

LPO levels were significantly higher in the kidneys of rats in the BPA group than in the control group (*p* < 0.01).This increase was significantly reduced in the BPA + G50 and BPA + G200 groups compared with the BPA group (*p* < 0.05 and *p* < 0.01, respectively) (Fig. 2D).

#### Kidney CrPr content

The results revealed a significant elevation in CrPr content in the BPA group compared with the control group (*p* < 0.001). This increase was significantly reduced in the BPA + G50 and BPA + G200 groups compared with the BPA group (*p* < 0.001 for both) (Fig. 2E).

#### Kidney antioxidants

**Figure 3:**
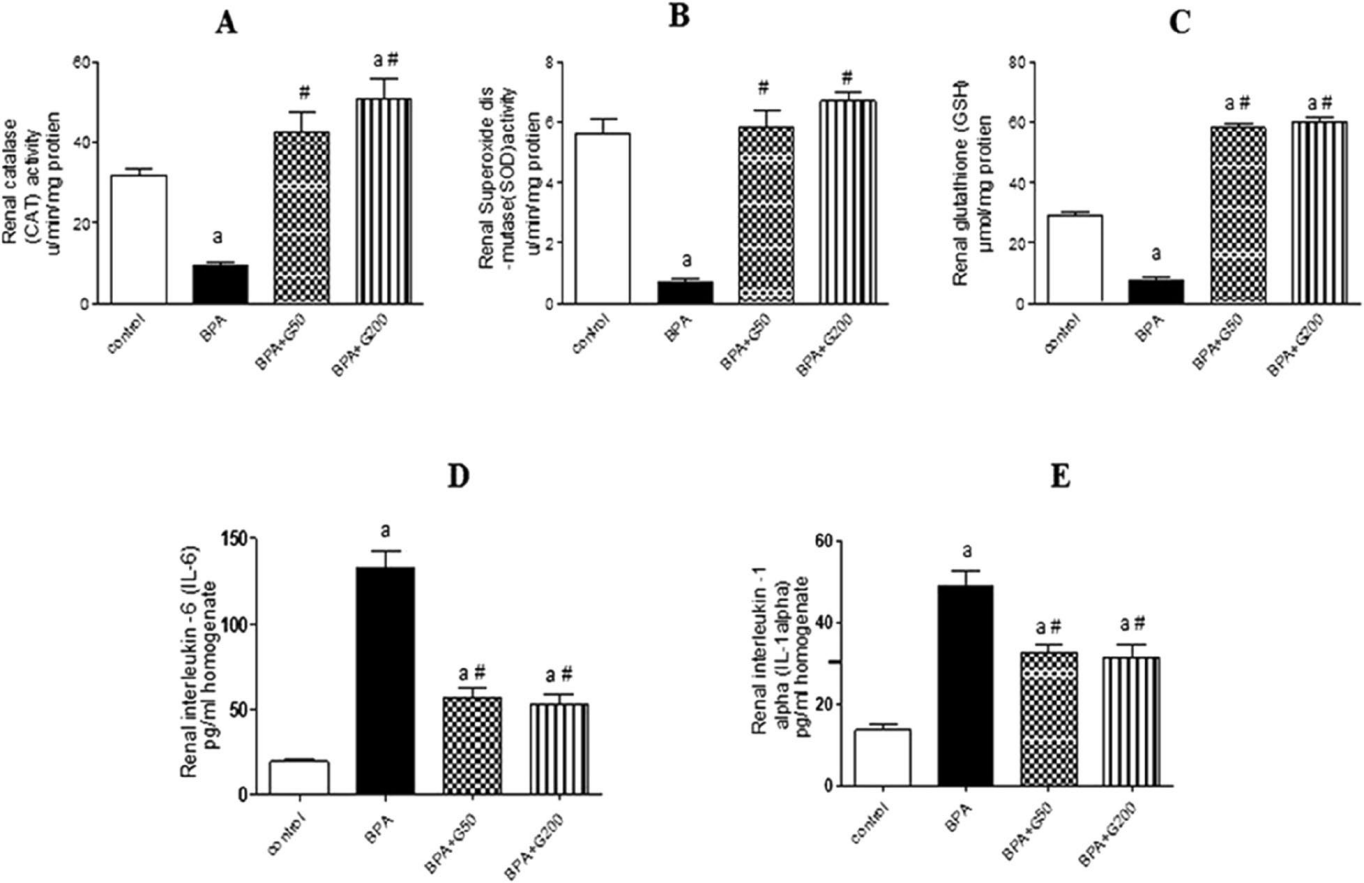
Ameliorative effects of GA (50 mg and 200 mg/kg bwt) on some antioxidants in the kidney, catalase activity (CAT) (**A**), superoxide dismutase activity (SOD) (**B**), and glutathione level (GSH) (**C**) in rats-treated with BPA. Columns with different superscripts are significantly different at *p* < 0.05 (^a^versus control and ^#^versus BPA). GA (50 mg and 200 mg/kg bwt) ameliorates the effects of BPA on some pro-inflammatory cytokines in the kidney, interleukin-6 (IL-6) (**D**) and interleukin-1α (IL-1α) (**E**). Columns with different superscript are significantly differences at *p* < 0.05 (^a^versus control and ^#^versus BPA).

Statistically, in the kidneys of BPA-treated animals, a significant inhibition of catalase (CAT) and superoxide dismutase (SOD) activities by (*p* < 0.01 and *p* < 0.001) respectively, in comparison to the control animals was prominent. However, this inhibition of CAT and SOD in the BPA group was significantly reversed in the BPA + G50 and BPA + G200 groups compared with the BPA group (*p* < 0.001 for both). CAT activity was significantly increased in the BPA + G200 group compared with the control group (*p* < 0.001) (Fig. 3A,B). In addition, GSH levels were significantly lower in the BPA group compared with the control group (*p* < 0.001). However, this decrease was significantly reversed in the BPA + G50 and BPA + G200 groups compared with the BPA group (*p* < 0.001 for both) (Fig. 3C).

#### Kidney proinflammatory cytokines (rIL-6 and IL-1α)

The levels of rIL-6 and IL-1α were significantly increased in the BPA group compared with the control group (*p* < 0.001). However, the levels of rIL-6 and IL-1α were significantly decreased in the BPA + G50 and BPA + G200 groups compared with the BPA group (*p* < 0.001 for both) (Fig. 3D,E).

### Morphology

#### Histological analysis

##### Hematoxylin and eosin (H&E) and toluidine blue staining

**Figure 4:**
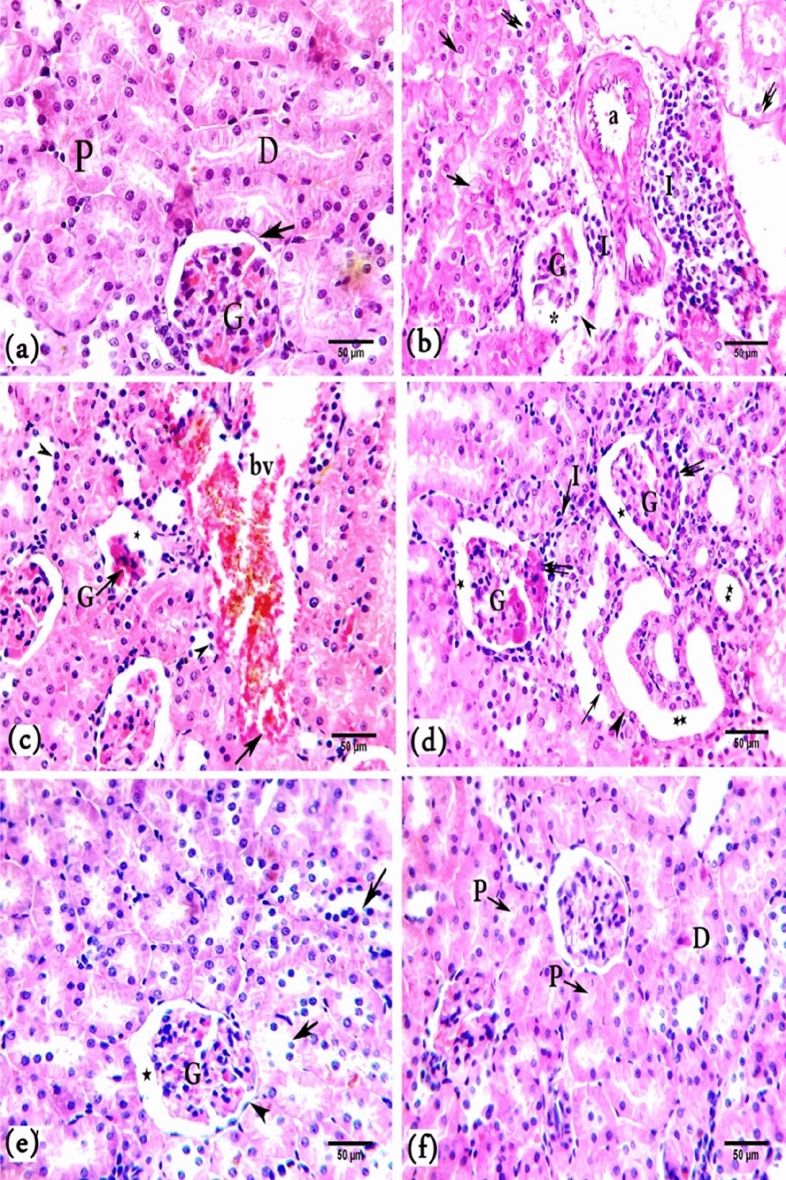
A photomicrograph of kidney cortex sections of rats (**a**). Control rats showing normal glomeruli (G), Bowman’s capsule (arrow), proximal convoluted tubule (P), and distal convoluted tubule (D). (**b**) BPA-treated rats showing the kidney tissue lost its architecture, and the lumen of some renal tubules seemed to be obliterated. leukocytic infiltration (I), dilated blood vessel (**a**), atrophy of the glomerulus (G), large capsular space (_*_), damaged parietal layer of Bowman’s capsule (head arrow), vacuolation (↑) and necrosis (↑↑) of most tubular epithelial cells. (**c**) BPA-treated rats with dilated and congested blood vessels (bv) with wall destruction (↑), glomerular atrophy (G), large capsular space (*), and tubular necrosis (head arrows) (**d**) BPA-treated rats showing flattened tubular epithelial lining (head arrow), leaving a large lumen (_**_), tubular necrosis (↑), leukocytic infiltration (I), lobulated glomeruli (G), wide capsular space (*), and destruction of their capsules (↑↑). (**e**) BPA + G50-treated rats showing few destructed tubular cells (↑), capsular space (_*_), normal glomerulus (G), and Bowman's capsule (head arrow). (**f**) BPA + G200-treated rats showing normal renal corpuscle and renal tubules (P&D) structures. (H&E stain, × 40).

In H&E staining, a normal kidney cortex structure was observed in control rats (Fig. 4a). However, several histopathological changes in the kidney were observed in the rats in the BPA group compared with those in the control group. The kidney tissue lost its architecture, and the lumen of some renal tubules seemed to be obliterated. Also, the alterations were seen in the Malpighian corpuscles, indicating damaged glomerular capillaries that resulted in a broad capsular gap (Fig. 4b,c). Damage to the epithelial lining and its brush borders was observed in several proximal and distal convoluted tubules (Fig. 4b). Also, necrotic cells and large lumens were observed in these tubules owing to flattening of the epithelial lining (Fig. 4d). Infiltrated leukocytes and congested blood vessels were observed (Fig. 4b,c). However, improvement in the cortical kidney structure in terms of renal tubules and Malpighian corpuscles was observed in the BPA + G50 and BPA + G200 groups compared with the BPA group (Fig. 4e,f). The development in gallic acid groups demonstrated by the majority of tubular cells having brush borders and normal-sized lumens.

**Figure 5:**
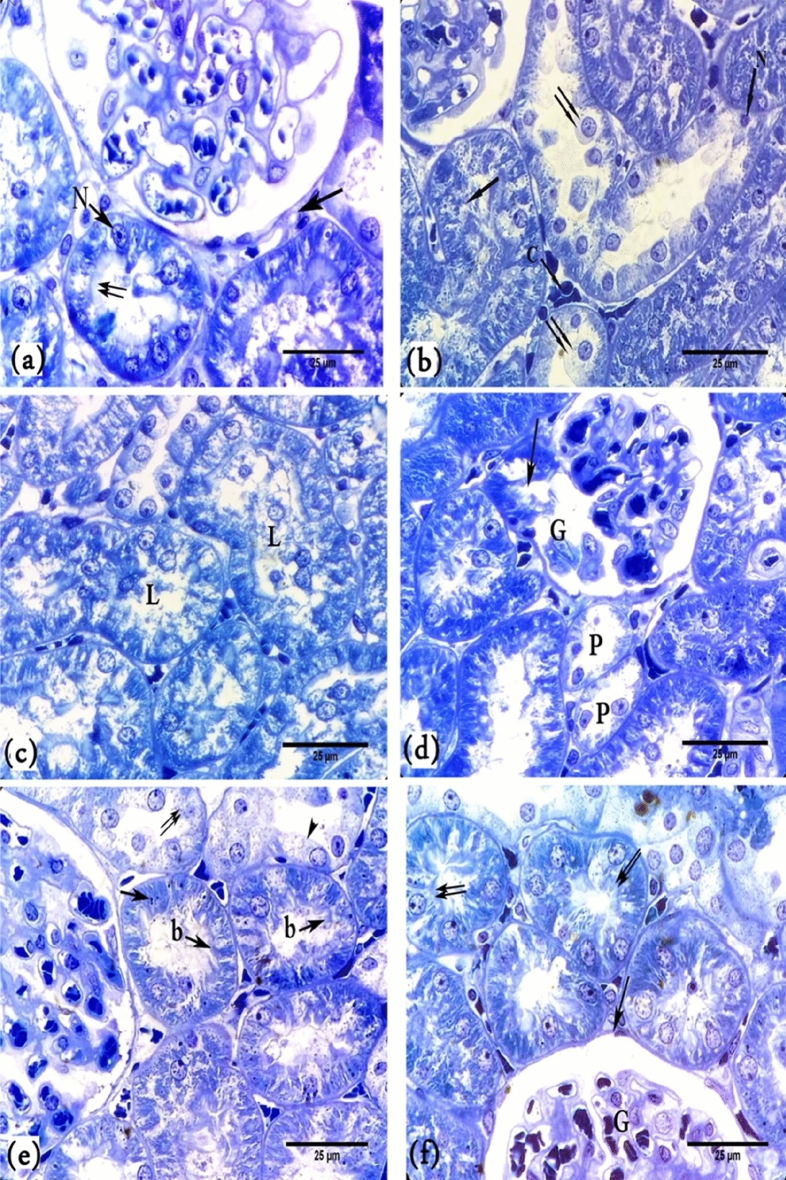
A photomicrograph of kidney cortex in semithin sections of rats (**a**). Control rat showing the proximal tubule exhibits apical brush borders (↑↑), rounded vesicular nuclei with prominent nucleoli (N), peritubular capillaries (↑) and a normal renal corpuscle. (**b**) BPA-treated rats showing hydropic degeneration of most tubular cells (↑↑), destruction of brush borders (↑), pyknosis of many nuclei (N), and congestion of peritubular capillaries (C). (**c**) BPA-treated rats showing exfoliated cells in the lumens (L) (**d**). BPA-treated rats showing necrosis of some proximal tubules cells (P), split of the glomerular tufts of capillaries (G) and their capsule (↑). (**e**) BPA + G50-treated rats have more advanced renal corpuscle; many cortical tubules have apical brush borders (**b**), and their cytoplasm contains basophilic granules (↑), a few hydropic degenerating cells (head arrow), and other cells have been completely destroyed (↑↑). (**f**) BPA + G200-treated rats showing normal renal corpuscle (G), intact renal corpuscle (↑) and renal tubules exhibiting apical brush borders (↑↑). (Toluidine blue stain, × 100).

In toluidine blue staining, semithin sections of the control group revealed that the cytoplasm of the cells of the proximal tubule exhibited rounded vesicular nuclei with prominent nucleoli. The apical cell membrane showed brush borders (Fig. 5a)**.** However, destruction of most proximal and distal convoluted tubular cells was observed in the BPA group. These destroyed tubular cells exfoliated into the lumen. Also, some of the tubular cells showed hydropic degeneration (Fig. 5b,c). Splitting of glomerular tufts and their capsule was observed (Fig. 5d). In the BPA + G50 and BPA + G200 groups, many cortical tubules exhibited apical brush borders, and their cytoplasm contained basophilic granules. Rounded vesicular nuclei with prominent nucleoli were also observed. However, only a few cortical tubular cells showed hydropic degeneration. Renal corpuscles in these groups appeared approximately similar to those in the control group (Fig. 5e,f).

##### Masson’s trichrome stain

**Figure 6:**
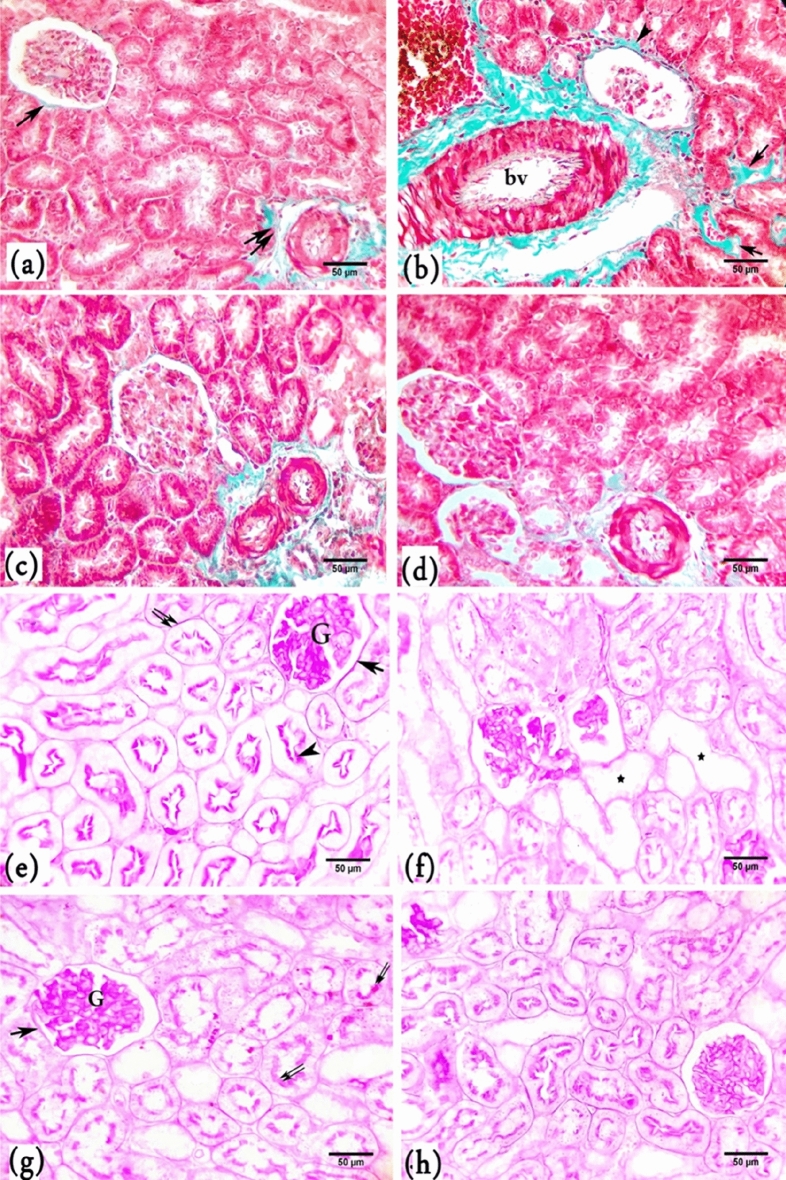
A photomicrograph of kidney cortex sections of rats showing; (**a**–**d**) Masson’s trichrome stain: (**a**) Control rats showing fine collagenous fibres surrounding Bowman's capsule (↑) and the blood vessels (↑↑). (**b**) BPA-treated rats showing large amounts of collagenous fibres around the dilated, thickened wall of the blood vessel (bv), between the renal tubules (↑) and around Bowman’s capsule (head arrow). (**c**) BPA + G50-treated rats showing a moderate amount of collagenous fibres around the Bowman’s capsule, between the renal tubules, and around renal blood vessels. (**d**) BPA + G200-treated rats had less collagenous fibres around the Bowman’s capsule, between the cortical tubules, and around the blood. (**e**–**h**) PAS reaction: (**e**) Control rats showing a strong PAS + ve reaction of polysaccharides in glomerular tufts of capillaries (G), Bowman’s capsule (↑), brush borders (head arrow), and the basement membrane of renal tubules (↑↑). (**f**) BPA-treated rats showing a decrease in the carbohydrate content of many renal tubules (_*_) and Malpighian corpuscles. (**g**) BPA + G50-treated rats showing positive PAS reactions in many renal tubules (↑↑), Bowman’s capsule (↑) and glomerular tufts of capillaries (G). (**h**) BPA + G200-treated rats exhibited strong PAS + ve reactions in the majority of renal tubules, Bowman’s capsule, and glomerular capillary tufts (× 40).

Few and fine collagen fibres were observed surrounding the Bowman’s capsule, the blood vessels, and the basal lamina of the renal tubules in the control group (Fig. 6a). The amount of collagen fibres surrounding the Bowman’s capsule and renal blood vessels and between the kidney tubules was increased in the BPA group (Fig. 6b). In the BPA + G50 and BPA + G200 groups, an obvious reduction in the amount of collagen fibres in the tissue was noted compared with the BPA group. This amount was approximately similar to that of the control (Fig. 6c,d).

#### Histochemical analysis

##### PAS

The polysaccharide content of the kidney cortex in the control group was represented by a deep purple color in the brush borders, the basement membranes of the renal tubules, the Bowman’s capsule, and the glomerular tufts of capillaries (Fig. 6e). A decrease in the PAS reaction was observed in the brush borders of renal tubules and the Malpighian corpuscles in the BPA group compared with the control group (Fig. 6f). An increase in the polysaccharide content in the renal tubules and Malpighian corpuscles was observed in the BPA + G50 and BPA + G200 groups compared with the BPA group. The polysaccharide content in the BPA + G50 and BPA + G200 groups appeared more or less similar to that in the control group (Fig. 6g,h).

#### Morphometric analysis of the histological and histochemical sections

##### Morphometric analysis of the histological sections (H&E staining)

- Glomerular space and capsular space areas

**Figure 7:**
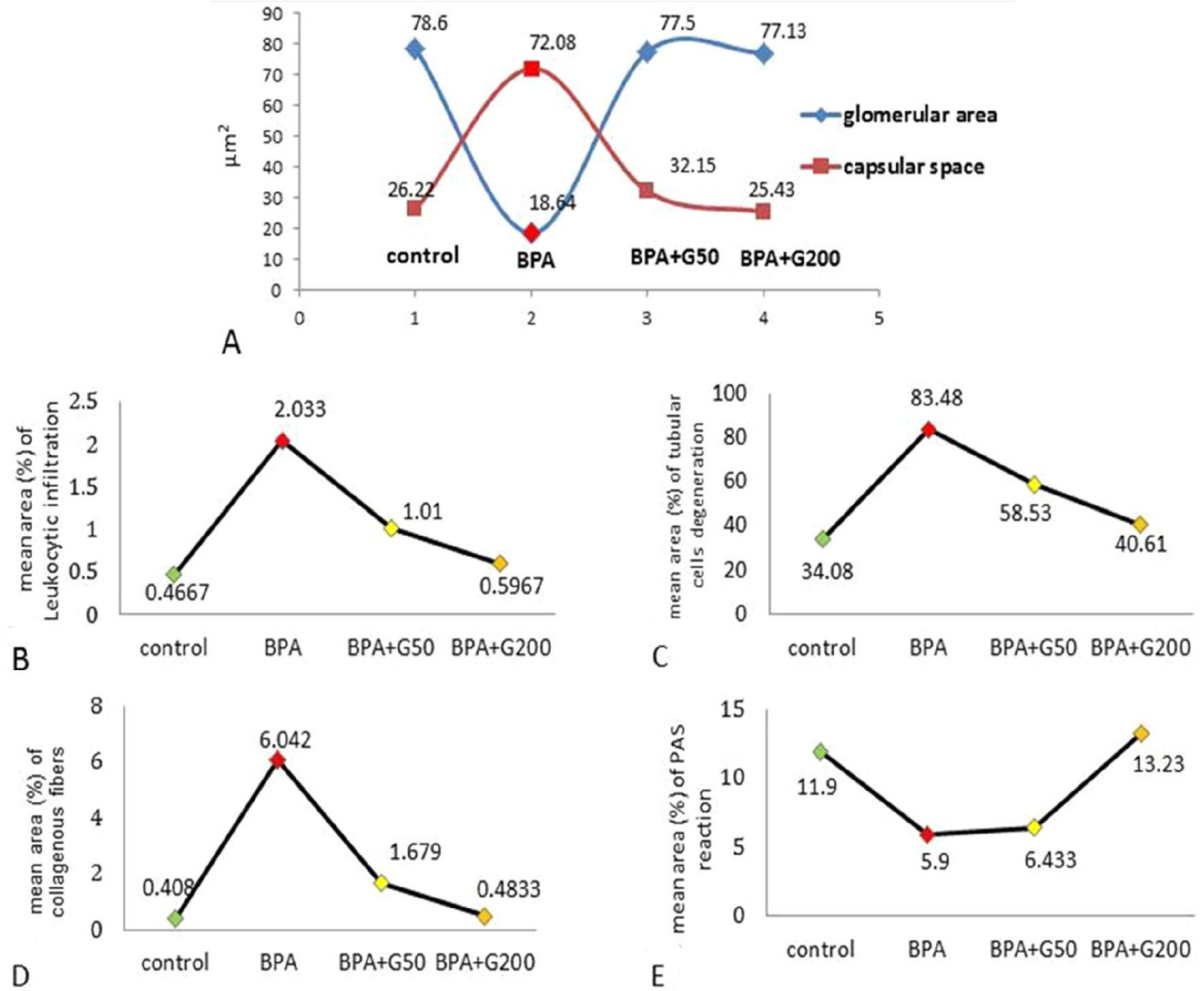
Mean morphometric analysis of kidney sections in control and treated groups of rats. (**A**) glomerular area and capsular space area; (**B**) leukocytic infiltration area of kidney sections; (**C**) tubular cell degeneration area in control and different treated groups of rats; (**D**) mean area percentage of collagenous fibers; and (**E**) mean area percentage of PAS reaction intensity.

As shown in Fig. 7A and Table 1, compared with the control group, the glomerular area was significantly decreased by 76.3% in the BPA group (*p* < 0.001). In the BPA + G50 and BPA + G200 groups, the percentage of the glomerular area increased significantly compared with the BPA group (*p* < 0.001) and was similar to that in the control group, with no significant differences. Further, there was no significant difference between the BPA + G50 and BPA + G200 groups.

**Table 1:** Mean value of morphometric analysis of glomerular area and capsular space area of kidney sections, increasing (%) or decreasing (%) in control and different treated groups of rats.

Groups	Measurements					
	Glomerular area	Increasing or decreasing %	%	Capsular space area	Increasing or decreasing %	%
Control	78.60 ± 1.78^a^			26.22 ± 2.53^a^		
BPA	18.64 ± 2.24^b^***	Dec. versus control	76.3	72.08 ± 6.52^b^***	Inc. versus control	174.9
BPA + G50	77.50 ± 3.84^ac^***	Dec. versus control	1.4	32.15 ± 1.56^ac^***	Inc. versus control	22.6
		Inc. versus BPA	315.8		Dec. versus BPA	55.4
BPA + G200	77.13 ± 1.37^ac^***	Dec. versus control	1.9	25.43 ± 1.67^ac^***	Dec. versus control	3.0
		Inc. versus BPA	313.8		Dec. versus BPA	64.7

Data are present as means ± SE. Values in the same column with unlike superscript letters.

^a^Control.

^b^Versus control.

^c^Versus BPA.

***Significance when *p* < 0.001.

As indicated in Table 1, the BPA group's capsular space area significantly increased by 174.9% when compared to the control group (*p* < 0.001).The BPA + G50 and BPA + G200 groups showed a decrease in the capsular space percentage compared with the BPA group (*p* < 0.001), but these groups showed no significant differences compared with the control group. Further, there was no significant difference between the BPA + G50 and BPA + G200 groups (Fig. 7A).

Based on these findings, it appears that gallic acid restores the normal size and structure of Malpighian corpuscles. As a result, it presumably gets the Malpighian corpuscles ready to resume their function in a healthy and normal manner.

b. Mean area of leukocyte infiltration

In Table 2, morphometric analysis revealed a 331.9% increase in the leukocyte infiltration areas in the BPA group compared with the control group (*p* < 0.001). In the BPA + G50 and BPA + G200 groups, there was a significant decrease in the percentage of the mean leukocyte infiltration area compared with the BPA group (*p* < 0.001). Compared with the control group, the BPA + G50 group showed significant differences in the percentage of the mean leukocyte infiltration area (*p* < 0.05), whereas the BPA + G200 group showed no significant differences. A significant difference was found between the BPA + G50 and BPA + G200 groups (*p* < 0.05) (Fig. 7B). These results demonstrated that large doses of gallic acid are capable of enhancing the kidney damage brought on by BPA.

c. Mean area of tubular cell degeneration

**Table 2:** Mean area of morphometric analysis of tubular cells degeneration and leukocytic infiltration of kidney sections, increasing (%) or decreasing (%) in control and different treated groups of rats.

Groups	Measurements					
	Tubular cells degeneration	Increasing or decreasing %	%	Leukocytic infiltration	Increasing or decreasing %	%
Control	34.08 ± 1.35^a^			0.47 ± 0.03^a^		
BPA	83.48 ± 2.41^b^***	Inc. versus control	145.0	2.03 ± 0.19^b^***	Inc. versus control	331.9
BPA + G50	58.53 ± 4.89^b^** ^c^***	Inc. versus control	71.7	1.01 ± 0.15^b^* ^c^***	Inc. versus control	114.9
		Dec. versus BPA	29.9		Dec. versus BPA	50.3
BPA + G200	40.61 ± 3.45^ac^*** ^d^**	Inc. versus control	19.2	0.60 ± 0.08^ac^*** ^d^*	Inc. versus control	27.7
		Dec. versus BPA	51.4		Dec. versus BPA	70.4

Data are present as means ± SE. Values in the same column with unlike superscript letters.

^a^Control.

^b^Versus control.

^c^Versus BPA.

^d^Versus BPA + G50.

*Significance when *p* < 0.05.

**Significance when *p* < 0.01.

***Significance when *p* < 0.001.

In the BPA group, tubular cell degeneration increased by 145% in comparison with the control group. The increase was statistically significant (*p* < 0.001). A statistical analysis of the two GA-treated groups showed a significant improvement (restoration of degenerated cells) (*p* < 0.001) compared with the BPA group. There were significant differences between the BPA + G50 group and the control and BPA + G200 groups (*p* < 0.01), as shown in Table 2 and Fig. 7C. Both dosages of gallic acid increase (restore) deteriorated cells' capacity to function more effectively.

##### Morphometric analysis of collagen fibre intensity

When compared to the control and GA-treated groups, the BPA group had a greater amount of collagen fibers. This increase was significant and was 1373.2% higher compared with the control group (*p* < 0.001). Rats in the BPA + G50 and BPA + G200 groups showed a decrease in the amount of collagen fibers compared with those in the BPA group (*p* < 0.001). Compared with the control group, the intensity of the collagen fibres in the BPA + G50 group significantly increased (*p* < 0.05), but no significant difference was noted in the BPA + G200 group. The intensity of collagen fibres was decreased to a greater extent in the BPA + G200 group than in the BPA + G50 group (Table 3 and Fig. 7D). Gallic acid therefore demonstrated its capacity to reduce the severity of fibrotic alterations brought on by BPA.

**Table 3:** Mean area of morphometric analysis of collagenous fibers and PAS reaction intensity of kidney sections, increasing (%) or decreasing (%) in control and different treated groups of rats.

Groups	Measurements					
	Intensity collagenous fibers	Increasing or decreasing %	%	Intensity of PAS reaction	Increasing or decreasing %	%
Control	0.41 ± 0.04^a^			11.90 ± 1.00^a^		
BPA	6.04 ± 0.59 b***	Inc. versus control	1373.2	5.90 ± 0.71^b^***	Dec. versus control	50.4
BPA + G50	1.68 ± 0.11^b^* ^c^***	Inc. versus control	309.8	6.43 ± 0.15 b***	Dec. versus control	46.0
		Dec. versus BPA	72.2		Inc. versus BPA	9.0
BPA + G200	0.48 ± 0.03^ac^*** ^d^*	Inc. versus control	17.1	13.23 ± 0.61^ac^*** ^d^***	Inc. versus control	11.2
		Dec. versus BPA	92.1		Inc. versus BPA	124.2

Data are present as means ± SE. Values in the same column with unlike superscript letters.

^a^Control.

^b^Versus control.

^c^Versus BPA.

^d^Versus BPA + G50.

*Significance when *p* < 0.05.

***Significance when *p* < 0.001.

##### Morphometric analysis of the PAS reaction intensity

Morphometric analysis of PAS reaction intensity in the BPA group revealed a significant decrease of 50.4% compared with the control group (*p* < 0.001). This depletion was not significantly reversed in the BPA + G50 group. However, the PAS reaction intensity was significantly decreased in the BPA + G50 group compared with the control group (*p* < 0.001). Although the depletion of PAS reaction intensity was significantly reversed in the BPA + G200 group compared with the BPA group (*p* < 0.001), no significant difference was noted between the BPA + G200 and control groups. Thus, the depletion of polysaccharide content was counteracted significantly more in the BPA + G200 group than in the BPA + G50 group (*p* < 0.001) (Table 3 and Fig. 7E). These findings highlight the importance of gallic acid in restoring polysaccharide levels. Gallic acid was more effective when given in high doses than low doses.

#### Electron microscopy (ultrastructure study)

**Figure 8:**
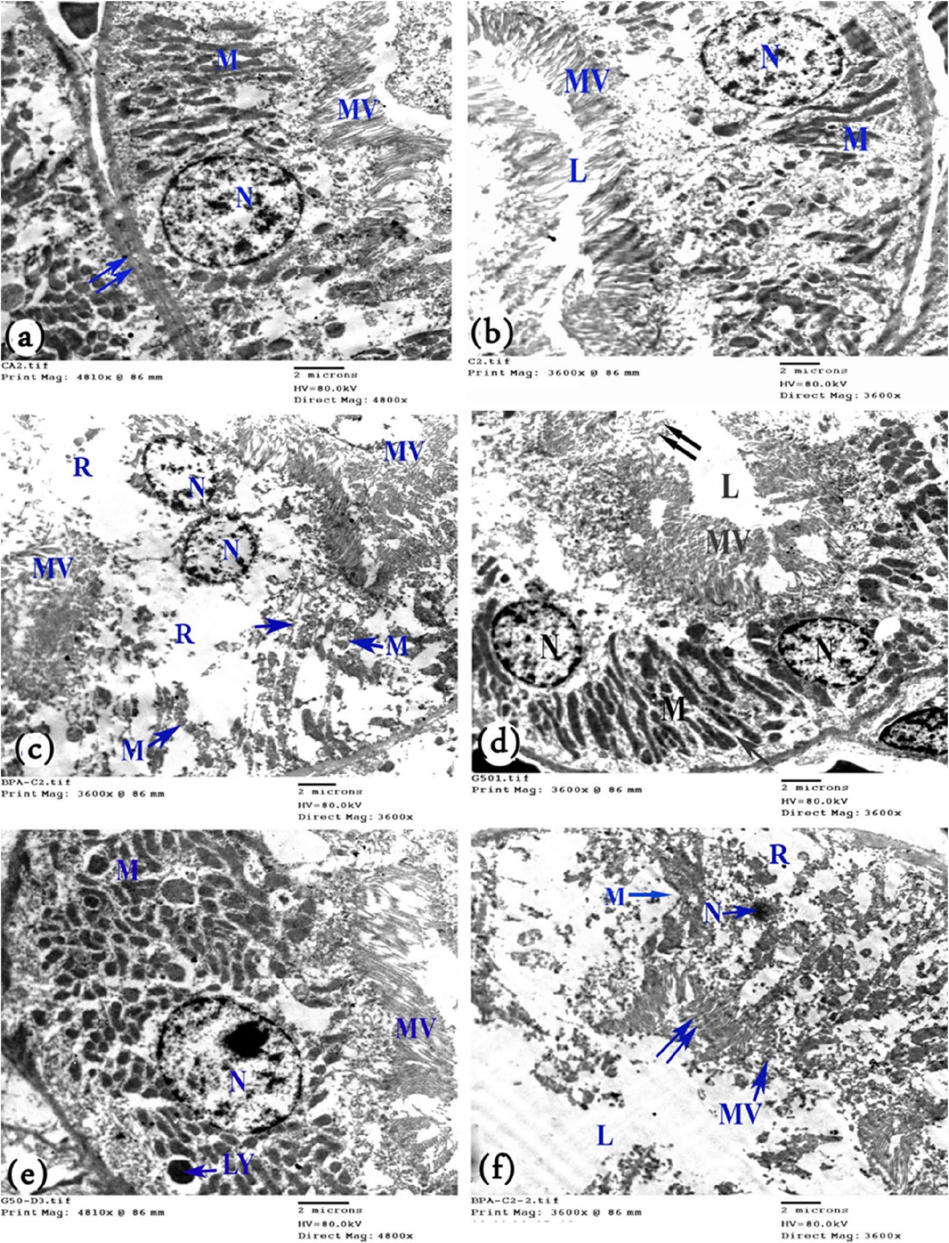
Electron micrograph of a cell in the proximal convoluted tubule of a rat’s kidney. (**a**) Control rats show a rounded basal euchromatic nucleus (N), numerous mitochondria (M) present within basal membrane infoldings, apical microvilli (MV), and basement membrane (↑↑). (**b**) Control rats showing narrow, clear lumen (L). (**c**) BPA-treated rats with rare cytoplasm (R) and nuclei that lost their basal localization and appeared in the apical part of the cell (N), as well as destructed mitochondria (M), basal infoldings (↑), and apical microvilli of the two adhering cells (MV). (**d**) BPA-treated rats showing a small nucleus (N), destructed microvilli (MV), disappearance of the basal infoldings and destroyed mitochondria (M), rarified cytoplasm (R), and the lumen filled with destructed organelles (L). Few microvilli are still present (↑↑). (**e**) BPA + G50-treated rats and (**f**). BPA + G200-treated rats showing a normal nucleus (N) with a prominent nucleolus, numerous polymorphic mitochondria (M) between the intact basal infoldings, intact microvilli (MV), and a narrow clear lumen (L). Note: lysosomes (LY).

The cells of the proximal tubules were cuboidal in shape, and the apical surfaces showed numerous microvilli in the control group rats. The basal membrane showed basal infoldings with numerous mitochondria of different sizes and shapes. The nuclei were large, rounded, and euchromatic with peripheral clumps of heterochromatin (Fig. 8a,b). The proximal tubule exhibited a narrow, clear lumen (Fig. 8b). In the BPA-group rats, the apical cell membrane showed destructed microvilli and their remnants. Damaged mitochondria were observed between widely destructed basal infoldings, and degeneration of cell organelles was noted, leaving large areas of rarified cytoplasm. The nuclei appeared small with irregular nuclear membranes. Destructed organelles were noted in wide lumens (Fig. 8c,d).

Electron microscopy analysis of the two GA-treated groups revealed a notable improvement in the proximal tubular cells. This improvement was represented by intact microvilli in the proximal tubular cells and regular basal infoldings enclosing numerous polymorphic mitochondria. In addition, the nuclei appeared euchromatic, and narrow, clear lumens of proximal convoluted tubules were observed. The kidney cortex revealed few lysosomes (Fig. 8e,f).

#### Immunohistochemical and morphometric analyses

**Figure 9:**
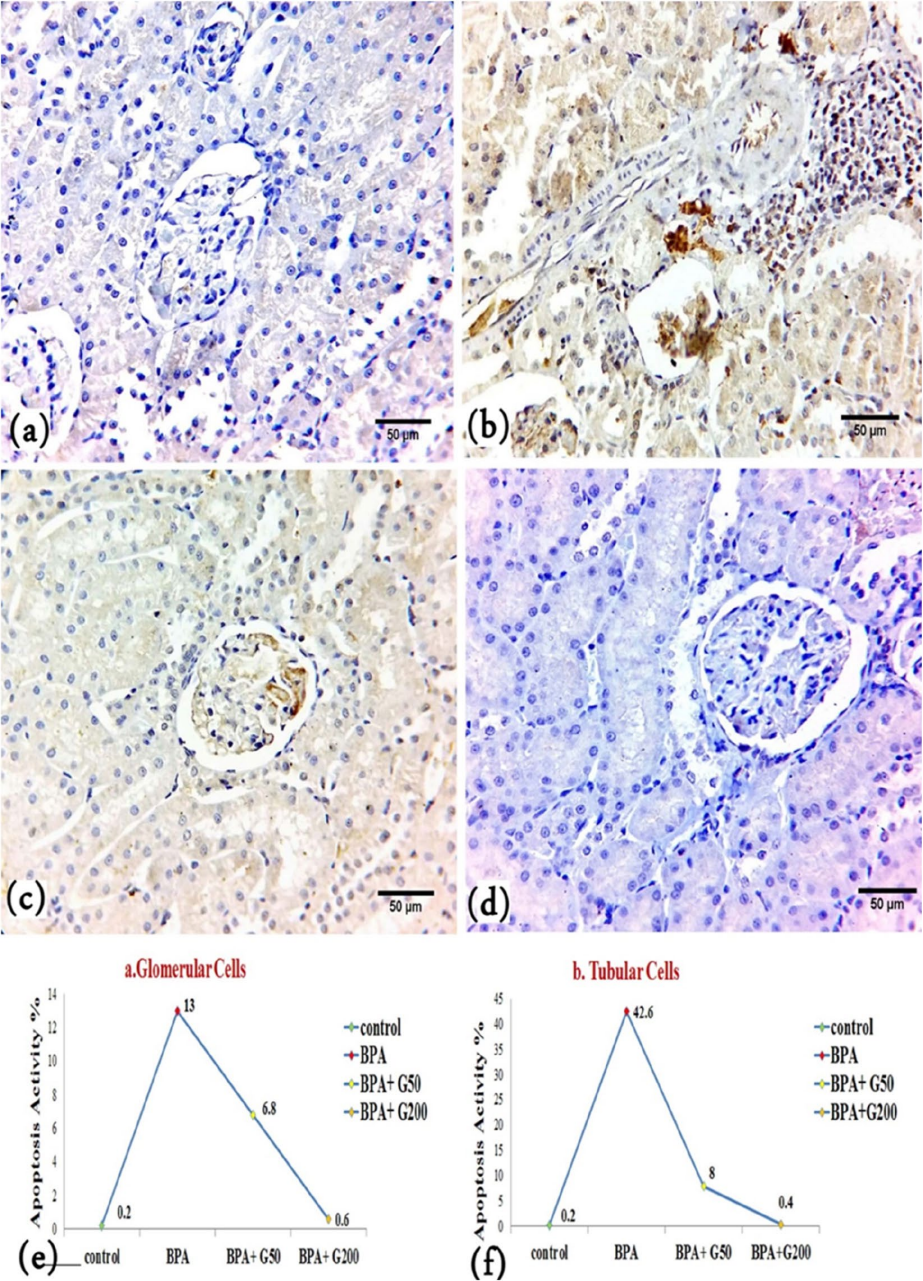
Immunohistochemistry findings of cytoplasmic Bax immunoreactivity in the different experimental groups (X40). (**a**) Control group showing a negative cytoplasmic Bax reaction in glomerular and tubular cells, (**b**) BPA-treated rats showing a strong positive cytoplasmic Bax reaction in leukocytic infiltration, glomerular and tubular cells. (**c**) BPA + G50-treated rats showed a decrease in the cytoplasmic Bax reaction in glomerular and tubular cells, and (**d**) BPA + G200-treated rats showed a negative cytoplasmic Bax reaction in both glomerular and tubular cells and appeared more or less similar to the control group. (**e**, **f**) Morphometric analysis of mean counts of positive Bax protein reaction of kidney sections in control and different treated groups of rats: (**e**) glomerular cells and (**f**) tubular cells.

Bax immunostaining was used to assess apoptosis in the kidney cortex epithelial cells. Negative cytoplasmic Bax immunoreactivity was noted in the control group (Fig. 9a). In the BPA group, most epithelial cells exhibited a strongly positive cytoplasmic Bax reaction in the form of a dark brown colour in the glomerular and tubular cells (Fig. 9b), with significant differences compared with the control group (*p* < 0.001) (Fig. 9e,f). In addition, there was a positive immune reaction in many infiltrated leukocytes (Fig. 9b). However, a decrease in the intensity of Bax immunoreactivity was observed in the glomerular and tubular cells in the BPA + G50 group compared with the BPA group (Fig. 9c). The decrease in the intensity of Bax immunoreactivity of glomerular cells in BPA + G50 was significant (*p* < 0.001) compared with that of the BPA group but increased significantly (*p* < 0.001) when compared with that of the control group (Fig. 9e). Bax immunoreactivity decreased significantly in the tubular cells (*p* < 0.001) of the rats in the BPA + G50 group compared with those in the BPA group (Fig. 9f). In the BPA + G200 group, the decrease in apoptotic activity was significant compared with the BPA group (*p* < 0.001) (Fig. 9d–f). Following the detrimental effects of bisphenol, gallic acid, an antioxidant, was able to return the cell to its normal state.

Table 4 compares the previous histopathological changes in kidney tissues in all treated groups to the control group.

**Table 4:** Summary of histological, and immunohistochemical findings and their scores in the kidney of control group and other groups in adult male albino rats.

Organ	Lesion	Control	BPA	BPA + G50 (50 mg/kg bwt)	BPA + G200 (200 mg/kg bwt)
Kidney	Atrophy of glomerular capillaries	−	+++	+	−
	Large capsular space	−	+++	+	−
	Tubular cells vacuolation	−	+++	−	−
	Tubular cells necrosis	−	+++	+	−
	Leukocytic infiltration	−	++	−	−
	Dilated blood vessels	−	++	−	−
	Congestion of blood vessels	−	++	−	−
	Widening of tubular lumens	−	++	−	−
	Cytoplasmic bax immunoreactivity	−	+++	+	−

Lesions were scored based on their severity as (− none, + mild, ++ moderate, +++ severe).

## Discussion

### Biochemical studies

The present study showed that BPA administration leads to a significant increase in the serum levels of urea, uric acid, and creatinine compared with the control. Our results corroborate those reported by Pal et al.^19^. El-Yamani^20^ reported that BPA causes hyperglycemia, which may be related to kidney damage and renal dysfunction and, consequently, increased serum creatinine, urea, and uric acid levels. The results of the present study showed that a significant increase in kidney function markers in BPA-treated rats was normalized after GA treatment at both doses. These results corroborate those reported by Dutta and Paul^21^, who carried out studies on rat livers.

In the present study, BPA intoxication resulted in a significant elevation in ROS levels compared with the control. Our results corroborate those reported by Manucha et al.^22^. This elevation in ROS levels may be a result of BPA-induced damage, mainly to mitochondrial peroxisomes. Consequently, it could be associated with the elevation of pro-oxidant parameters, such as rLPO and rCrPr, and the marked rise in ROS levels in response to BPA intoxication^23^. The marked rise in rROS levels observed in BPA-treated rats improved after GA treatment. Another study also reported that GA significantly reduces renal and cardiac ROS levels in BPA-treated rats^24^.

The data obtained herein showed that rNO levels significantly increased in response to BPA treatment compared with the control. This elevation in renal NO levels may be attributed to the disturbances in amino acid metabolism, possibly due to BPA treatment. Yıldırım^25^ demonstrated that in streptozotocin-treated rats, renal nitrite levels were increased owing to some disturbances in amino acid metabolism in diabetes, which may increase intrarenal NO production. Szentiványi et al.^26^ noted a chronic increase in plasma arginine vasopressin levels, which enhanced the protein expression of nitric oxide synthase in the renal medullary endothelium, resulting in a chronic increase in NO concentration in this region of the kidney to counteract the hypertensive effects of arginine vasopressin.

In the present study, NO levels normalized in BPA-treated rats after GA treatment at both doses. NO is related to many biological processes, such as vasodilatation^27^. GA relaxes the aortic rings through an endothelium-dependent pathway in rats, resulting in eNOS phosphorylation and potassium channel opening^28^. This suggests that GA could restore normal sNO levels in BPA-treated rats and might be associated with vasodilation, increased blood flow, and improved ischemia.

Peerapanyasut et al.^29^ reported an increase in MDA in the kidney and liver of BPA-treated rats, substantiating an alteration in mitochondrial redox homeostasis. In the present study, rLPO was significantly elevated in the kidney after BPA intoxication compared with the control. Khan et al.^30^ demonstrated that mitochondrial samples from rat livers treated with BPA had increased CrPr content. Similarly, the present study revealed a significant increase in rCrPr levels in response to BPA intoxication compared with the control. Consequently, BPA can induce nephrotoxicity. In addition, the elevated rCrPr levels may exacerbate kidney damage, which was revealed in the histological analysis in this study. Biochemical analysis of the rLPO levels in the BPA + G50 and BPA + G200 groups revealed normalization compared with the control. Our results corroborate those reported by Maurya et al.^31^, who reported that GA completely reversed LPO elevation in septic mice.

The present study showed that BPA intoxication induced significant inhibition of rCAT and rSOD activities and decreased rGSH levels compared with the control. These findings could be attributed to the overproduction of free radicals and their association with a marked increase in rCrPr levels, as shown in the present data. The effect of GA on improving rCrPr levels in the present study may be related to its ability to scavenge ROS formed due to BPA toxicity. In addition, all activities of renal enzyme reactions measured in the present study were increased. In another study, GA treatment significantly increased SOD activity and GSH levels compared with diazinon (insecticide) treatment in the renal tissues of rats^32^. Sheweita et al.^33^ reported that GA could restore renal CAT and SOD activities and GSH levels in tramadol-treated rats. Further, another study reported that GA significantly increased both renal and cardiac antioxidant levels in BPA-treated rats^24^.

In this study, BPA treatment resulted in a significant increase in rIL-6 and IL-1α levels compared with the control. The present results corroborate those reported by Moon et al.^34^ in BPA-treated rats. IL-6 can result in the improvement of renal disease^35^. Consequently, these findings could confirm that BPA-induced renal inflammation is strongly associated with an increase in both rIL-1α and rIL-6. In a rat cirrhosis model, GA prevented an increase in the levels of proinflammatory cytokines (IL-1α and TNFα) in the plasma and the depletion of the anti-inflammatory cytokine IL-4 resulting from chronic bile duct ligation^36^. GA significantly reduced both renal IL-6 and IL-1α levels in this study. These results may confirm the anti-inflammatory effects of GA on the kidneys of BPA-treated rats.

### Morphological study

The present study demonstrated that 30 days of oral administration of BPA results in multiple toxic morphological changes in the kidney tissues. The Malpighian corpuscles of the BPA-treated rats showed several lesions, including the splitting and atrophy of many glomerular tufts, loss of the glomerular epithelium, and widening of the capsular space. The morphometric analysis revealed a significant decrease in the glomerular area. Similar results were reported by Wahbby et al.^37^ and Ola-Davies et al.^38^ in BPA-treated rats. Herein, BPA resulted in a marked elevation in urea and creatinine levels. Ahmed et al.^39^ confirmed that the increased urea and creatinine levels in BPA-treated rats were associated with nephrotoxicity. In BPA-treated rats, atrophy of renal corpuscles with segmented glomerular tufts complicated by widened capsular spaces can exacerbate the impairment of the Bowman's capsule's filtration function^38^.

Examination of semithin sections revealed swollen and hydropic degeneration of proximal and distal convoluted tubular cells in BPA-treated rats. In response to injury, hydropic change is considered an early sign of cellular degeneration and may signify tissue hypoxia-related water accumulation in the cell. Hypoxia results from a decrease in aerobic respiration in mitochondria and ATP production^40^. Sangai and Verma^41^ confirmed a reduction in ATP production in the liver cells of BPA-treated mice. Shimizu et al.^42^ said that the necrosis could be caused by a lack of ATP, which led to cell death in the end.

Dogrul et al.^43^ examined poly (ADP-ribose) polymerase (PARP) in GA and BPA-treated cells. PARP is an enzyme involved in the DNA repair mechanism and apoptosis. They found a significant increase in PARP expression in GA HGF-1-treated cells. These results may be proof that GA has a positive effect on the DNA repair mechanism by increasing PARP expression in benign cells and decreasing replication in malignant cells. Gu et al.^44^ reported that GA in normal cells acts as a powerful antioxidant and has antiapoptotic effects, inhibiting dose- and time-dependent mitochondrial respiration, thereby resulting in decreased oxidative stress and its consequent harm.

Korkmaz et al.^45^ reported that BPA produces oxidative damage related to ROS in the brain and kidney. Hassan et al.^46^ explained that this oxidative damage could cause cell rupture and membrane damage in human erythrocytes. These findings corroborate those of the present study, which demonstrated that BPA causes degeneration and necrosis of both proximal and distal convoluted tubules. Moreover, there was exfoliation of destructed tubular cells in their lumens.

Leukocyte infiltration between renal tubules and around dilated, congested blood vessels in the renal cortex was observed in the BPA-treated group. The morphometric analysis also confirmed significantly larger leukocyte infiltration areas in the BPA-treated group compared with the control group. Our results were in agreement with the findings of Ola-Davies et al.^38^ in BPA-treated rat livers. Wetherill et al.^47^ reported that macrophages produce proinflammatory cytokines in BPA-treated animals. These mediators lead to the second phase of injury, involving endothelial cell adhesion molecules that intermediate the adhesion and transmigration of neutrophils from the vascular space into the parenchyma^48^. The proteases and oxidants released from these accumulations directly injure the vascular endothelial cells^49^.

GA showed a protective effect on BPA-induced leukocyte infiltration. The morphometric analysis of leukocyte infiltration supports this finding. These findings support those of Asci et al.^50^, who found that GA reduced tubular cell necrosis and decreased the expression of elevated inflammatory markers like TNF-, and that CRP changes were immunohistochemically significant in rats after methotrexate-induced kidney damage treatment.

In the current study, the amount of collagen fibres in the BPA-treated group was higher than in the control group.A similar observation was recorded by Kattaia and Abdel Baset^51^, who found increased collagen fibre deposition in the lungs of BPA-treated rats. Ramos et al.^52^ suggested that BPA causes fibroblast hyperplasia in different organs. The first step in the aetiology of organ fibrosis is the damage to the epithelia and their basement membranes. Many cell types, including inflammatory and immune cells, migrate to and proliferate in the affected areas and release abundant cytokines that cause further cell recruitment, inflammation, and eventually matrix remodeling. This finding demonstrates an overproduction of collagen^53^. Morphometric analysis in this study confirmed the increase of collagen fibers. This finding is in agreement with the results of Fadda et al.^54^, who reported similar findings in their study on BPA-treated rat kidneys.

Histological and morphometric analyses of the BPA + G200 group in the current study revealed that the amount of collagenous fibres in this group was lower than in the BPA group. However, this amount appeared more or less similar to that in the control group. This corroborates the findings of Fadda et al.^54^, who confirmed that the coadministration of green tea extract (a mixture of polyphenols) and BPA led to a significant decrease in the area of collagen deposition compared with BPA treatment alone. Polyphenols inhibited inducible NO synthase, Fas ligand, -actin smooth muscle, and desmin immunoreactivity in rat renal tissues, according to the authors. The results of previous studies are in line with those of the present biochemical analysis, which demonstrated that GA causes the depletion of NO. This could also explain the protective role of GA against vasodilatation in renal tissues.

The mechanism of blood vessel dilatation was explained by Palmer et al.^55^, who reported that NO is a small bioactive gas that was first identified as mediating arterial vasodilatation. The present biochemical study showed a substantial increase in NO production in the kidneys of BPA-treated rats, which might be related to blood vessel dilatation.

ROS and RNS (oxidative stress) are formed during normal metabolic activities in various biochemical reactions and cellular functions, such as mitochondrial respiration. Any excess of ROS- and RNS-induced deterioration of macromolecule structures (carbohydrates, proteins, lipids, and nucleic acids) leads to the loss of organ function^56^_**.**_ The increase in ROS levels in the BPA-treated rats in the present study resulted in a decrease in the polysaccharide content, suggesting loss of kidney function. The decrease in PAS reaction intensity in this group compared to the control group was also shown by the morphometric analysis.

Electron microscopy revealed the degeneration of cell organelles, leaving areas of rarified cytoplasm. El-Banhawy and Ganzuri^57^ illustrated that these changes could be due to the injuries to the lysosome membranes caused by many drugs. These membranes are destroyed due to their sensitivity to any pathologic effects and the release of their powerful enzymes, leading to the dissolution and degeneration of various cellular components.

Bax immunostaining showed a strong positive reaction in most of the epithelial cells of the kidney cortex in the BPA-treated group. This result was similar to that of Peerapanyasut et al.^58^, who found a significant increase in the Bax/Bcl-2 ratio in the kidneys of BPA-treated rats. Accordingly, Bosch-Panadero et al.^59^ demonstrated that apoptotic death in kidney tubular cells was observed in rats exposed to BPA. Olea-Herrero et al.^60^ also found that BPA could cause apoptotic death in podocytes in culture media. Kourouma et al.^61^ illustrated that the increase in LPO caused by BPA was related to caspase activity. This activation causes many apoptotic signals to be sent, which leads to apoptosis and hepatotoxicity in rat liver tissue.

In the present study, the two doses of GA were able to decrease cytoplasmic Bax immunoreactivity in the renal cortex tissue. Also, the morphometric analysis of Bax immunostaining confirmed the histological results obtained herein. Mard et al.^62^ reported a significant decrease in the protein expression of caspase-3 after GA treatment. Similarly, GA pretreatment in a model of human epithelial cells (HeLa cells) has been associated with a decrease in the increased activity of caspase-9 resulting from H_2_O_2_-induced oxidative stress, which ultimately causes apoptotic cell death^63^.

## Materials and methods

### Materials

#### Chemicals

BPA [4,4′-(propane-2,2-diyl)diphenol; 98% pure] was purchased from Alpha Dark English Company. GA (3,4,5-trihydroxybenzoic acid; 99.5% pure) was purchased from Oxfored Company. Sodium dodecyl sulphate (SDS), epinephrine, potassium dibasic, potassium monobasic, trichloroacetic acid, ethylene diamine tetraacetic acid (EDTA), 5,5-dithiobis-2-nitrobenzoic acid, hydrogen peroxide (H_2_O_2_), salfosalsylic acid, and thiobarbituric acid were purchased from Fluka Company. All other chemicals were of the highest available quality.

#### Experimental animals and ethics

##### Experimental animals

Forty healthy adult male Wistar albino rats (12 weeks old, mean weight 130 ± 30 g) were maintained under normal day/night conditions (12 h of light:12 h of darkness) at room temperature (25 °C ± 2 °C). They were maintained under standard laboratory care and provided food and tap water ad libitum throughout the experimental period. The animals were purchased from Assiut University Joint Animal Breeding, Assiut, Egypt.

The rats were allowed to acclimatize for 2 weeks before initiating the experiment. The rats were weighed and assigned to four groups according to their body weights to achieve approximately the same mean body weight for each group.

### Ethical approval

The experimental setup and animal handling procedures were approved by the Research Ethical Committee of the Faculty of Science, Assuit University, Assuit, Egypt. All experimental procedures were performed in accordance with the relevant ARRIVE guidelines and regulations. And all methods were performed in accordance with the relevant guidelines and regulations.

### Methods

#### BPA preparation

BPA grains (40 mg) were first dissolved in 1 ml of absolute ethanol (1% ethanol) and then dissolved in 100 ml of drinking water. According to Mahmoudi et al.^64^, ethanol can be used as a solvent for BPA. A standard working solutions of BPA was prepared by dilution of the stock standard solution with ethanol.

#### GA preparation

To avoid the loss of GA doses in the normal diet, GA powder was first dissolved in some drinking water and mixed with the food of the rats daily.

#### Experimental design

Male rats were employed in the current investigation as the standard since female rats could be unpredictable and exhibit varying responses to the same stimuli as a result of the hormone changes that occur throughout the female cycle.

The animals were randomly divided into two groups: Group 1 (control): 10 control rats received a normal diet and water. Group 2 (treated animals): 30 rats were treated with BPA. BPA was added to the drinking water provided ad libitum (tap water) (40 mg/100 ml), which was nearly equivalent to (40 mg/kg bwt) according to Wahbby et al.^37^. This group was then divided into three subgroups (10 animals each). Subgroup 1 was considered the positive control treated with BPA and designated as BPA; Subgroup 2 was administered with GA (G50; 50 mg/60 g diet) ad libitum to a normal diet equivalent to that of a previous study (50 mg/kg bwt) and designated as BPA + G50. Subgroup 3 was administered GA (G200; 200 mg/60 g diet) ad libitum to a normal diet equivalent to that of a previous study (200 mg/kg bwt) and designated as BPA + G200. The GA doses used in the present study were determined according to the study by Karimi-Khouzani et al.^65^.

For 30 days, the administrations were repeated daily. All experimental protocols on animals were performed according to the regulations of the Institutional Animal Care and Use Committee and were approved by Assiut University.

#### Experimental procedures

##### Blood collection

Blood samples were collected by cardiac puncture from each rat for haematological studies. The cardiac puncture was performed when the rats were deeply anesthetized; blood was drowning slowly according to the procedure described by Parasuraman et al.^66^. Blood that did not contain EDTA was used for the preparation and separation of serum after centrifugation at 3000 rpm for 5 min. Serum samples were kept at − 20 °C for subsequent biochemical analysis.

##### Preparation of kidney homogenate for biochemical assays

For the preparation of 10% w/v kidney homogenate for biochemical assays, 500 mg of kidney tissue was homogenized in 5 ml of phosphate buffer (0.1 M, pH 7.4) using a homogenizer (IKA Yellow Line DI 18 Disperser, Germany). The homogenates were centrifuged at 5000 rpm for 30 min at 4 °C, and the supernatant cytosols were kept frozen at − 20 °C for subsequent biochemical analysis.

##### Preparation of kidney homogenate for immune assays

For preparing kidney homogenates for immune assays, 500 mg of kidney tissue were used. Tissue homogenates were prepared using RIPA buffer [20 mM Tris–HCl, pH 7.5; 120 mM NaCl; 1.0% Triton X-100; 0.1% SDS; 1% sodium deoxycholate; 10% glycerol; 1 mM EDTA; and 1% protease inhibitor cocktail (Roche; Basel, Switzerland)].

##### Tissue preparation

After 30 days of treatment, the rats were subsequently anaesthetized with urethane (1.25 g/kg, i.p.) and sacrificed by cervical dislocation^67^, and their kidneys were excised immediately. Kidney pieces were divided into two parts: one part was used for homogenization, and the other was fixed in formol acetic alcohol for histological and histochemical analyses.

#### Biochemical analyses

##### Kidney function

Tietz’s^68^ method was used to measure serum creatinine, and Tietz’s^69^ method was used to measure serum urea and uric acid.

##### Free radical content in the kidney

- Reactive oxygen species (ROS) and Nitric oxide (NO) levels

ROS levels were estimated according to the method described by Kalyanaraman et al.^70^. Nitric oxide (NO) levels in the kidney and serum were colorimetrically measured as nitrite concentrations according to the method described by Ding et al.^71^.

b. Lipid peroxidation (LPO) and Carponyl protein (rCrPr)

LPO was estimated using the method described by Ohkawa et al.^72^. Carponyl protein (rCrPr) was estimated according to the method described by Stadtman and Levine^73^. LPO and (rCrPr) are regarded as indicators of oxidative stress.

##### Antioxidant determinations in the kidney

Catalase (CAT) activity was measured according to the method described by Beers and Sizer^74^. Superoxide dismutase (SOD) activity was estimated according to the method described by Misra and Fridovich^75^. Also, glutathione (GSH) was estimated according to the method described by Ellman^76^.

##### Proinflammatory cytokine levels in the kidney

The levels of interleukin 6 (IL-6) and interleukin 1α (IL-1α) were measured via enzyme-linked immunosorbent assay using a Bio-Plex Cytokine Assay Kit (Bio-Rad, Hercules, CA, USA) according to the manufacturer’s instructions.

##### Total protein assay

The total protein levels were determined according to the method described by Lowry^77^.

#### Preparation of histological sections

##### Histological and histochemical analyses

*Light microscopy* Formol acetic alcohol was used to fix small kidney specimens. Before further processing, the fixed samples were thoroughly washed in 70% ethanol (3 × 24 h) to remove the fixative. The samples were dehydrated in ascending grades of alcohol (80%, 90%, 100I, 100% II), cleared in methyl benzoate, and then embedded in paraffin wax for three hours^78^. Sections were cut 4–5-µm thick and stained with hematoxylin and eosin^79^ (H&E) for general histology and Masson’s trichrome stain^80^ for detection of collagen fibers. Periodic acid–Schiff (PAS) staining was used to detect polysaccharide content according to the method described by McManus^81^. Some paraffin sections were used for immunohistochemical analysis. All stained sections were examined under light microscopy; a Leitz Dialux 20 microscope provided with a digital camera captured the images.

##### Immunohistochemical analysis

Bax is proapoptotic and functions as a promotor of apoptosis^82^. As a result, the expression of Bax in the current study assessed apoptosis in kidney cortex epithelial cells.

Immunohistochemical staining for Bax, deparaffinization, and dehydration were performed with xylene and alcohol. After washing the sections with distilled water, antigen retrieval was performed using a microwave-based method, followed by incubation in a protein blocking solution.

For Bax protein, rabbit polyclonal anti-rat antibody (1:1000 to 1:2000 v/v) was used (from immunoglobulin (Ig) fractions, diluted in PBS, pH 7.6, containing 1% BSA and 0.09% sodium azide). Subsequently, the sections were incubated for 1 h with 1:200 (v/v) biotinylated goat anti-rabbit antibody (Dako A/S, Copenhagen, Denmark) and peroxidase-conjugated streptavidin (Dako A/S). DAB (3,3′-diaminobenzidine) was stained at room temperature with hematoxylin for counterstaining. Under a light microscope, the presence of dark brown precipitates was determined as positive expression or staining^83^. Images were captured using a Leitz Dialux 20 Microscope equipped with a digital camera.

##### Transmission electron microscopy

Small specimens were taken from the kidney after sacrificing the animals) and fixed in 5% cold glutaraldehyde for 24 h. The specimens were then washed four times for 20 min each in cacodylate buffer (pH 7.2) and post-fixed in cold osmium tetraoxide for two hours. Thereafter, the specimens were washed four times for 20 min each in cacodylate buffer. Dehydration was performed using increasing concentrations of ethyl alcohol (30%, 50%, and 70%), with each treatment lasting for 2 h. This was followed by two treatments each of 90% and 100% ethyl alcohol for 30 min. Embedding was performed in Epon 812. The embedded samples were kept in an incubator at 35 °C for 1 day, at 45 °C for another day, and at 60 °C for 3 days^84^. A LKB ultramicrotome (Germany) was used to prepare semithin sections of 0.5–1-μm thickness, which were then stained with toluidine blue, observed with a light microscope, and photographed. Ultrathin sections (50–80 nm) from selected areas of the trimmed blocks were taken and collected on a copper grid. The ultrathin sections were contrast stained with uranyl acetate for 10 min and with lead citrate for 5 min, after which they were examined via transmission electron microscopy (JEOL 100 CX; JEOL, Japan) and photographed at 80 kV in the Assiut University Electron Microscopy Unit.

#### Morphometric analysis

After the rat kidneys were histologically processed and stained, Digital images were obtained under 40 × magnification using a digital camera connected to a light microscope; a Leitz Dialux 20 Microscope provided with a digital camera captured the images.

Morphometric analysis was performed using the computerised image analysis software ImageJ version 6. Spatial calibration with an object micrometre was performed before each analysis. Five images were selected from each animal in each group. The following morphometric parameters were measured: glomerular area (µm^2^), capsular space area (µm^2^) of Malpighian corpuscles, areas of tubular cell degeneration (%), and leukocytic infiltration (%) stained with H&E. Collagen fibre intensity (%) and polysaccharide content (%) were measured. The mean counts of the Bax positive reaction areas in glomerular and tubular epithelial cells were measured.

#### Statistical analysis

A close comparison was made between the data obtained from the normal group and those from the treated groups. The comparison results were expressed as means standard errors. One-way analysis of variance was used to test the differences between the means, followed by the Student–Newman–Keuls test for multiple comparisons. These analyses were conducted using the Prism 5.0 package (Graph and Software, Inc., San Diego, USA) and Microsoft Excel. The threshold for statistical significance was set at *p* < 0.05, 0.01, or 0.001. The percent of stimulation (S%) or inhibition (I%) in the mean values was calculated as follows:

$${\text{I}}\;{\text{or}}\;{\text{S\% }} = \tfrac{{{\text{Mean}}\;{\text{control}}\;{\text{value}} - {\text{Mean}}\;{\text{treated}}\;{\text{value}}}}{{{\text{Mean}}\;{\text{control}}\;{\text{value}}}} \times {100}{\text{.}}$$

For different treatments as follows:

$${\text{I}}\;{\text{or}}\;{\text{S\% }} = \tfrac{{{\text{Mean}}\;{\text{value}}\;{\text{of}}\;{\text{treatment1}} - {\text{Mean}}\;{\text{value}}\;{\text{of}}\;{\text{treatment2}}}}{{{\text{Mean}}\;{\text{value}}\;{\text{of}}\;{\text{treatment1}}}} \times {100}{\text{.}}$$

## Conclusion

This study suggests that the use of GA restores the deleterious effects induced by BPA, possibly due to its antioxidant properties. The high dose of GA was more effective than the low dose. Hence, the consumption of nutrients rich in polyphenols (GA) could reduce the side effects and toxicity resulting from the use of any products containing BPA or other harmful substances. It is necessary to stay away from any products that contain BPA and use glasses or pottery to avoid the use of and injuries resulting from BPA as much as possible.

## Data Availability

The data that support the findings of this study are available from the corresponding author upon reasonable request.

## References

[1] Vandenberg LN, Maffini MV, Sonnenschein C, Rubin BS, Soto AM (2009). Bisphenol-A and the great divide: A review of controversies in the field of endocrine disruption. Endocr. Rev..

[2] Welshons WV, Nagel SC, vom Saal FS (2006). Large effects from small exposures. III. Endocrine mechanisms mediating effects of bisphenol A at levels of human exposure. Endocrinology.

[3] Michael, E. Sarah A. Vogel. *Is It Safe? BPA and the Struggle to Define the Safety of Chemicals*. xxi + 304 pp., illus., index. Berkeley: Univ. of California Press. 2013. *Isis* **105**(1), 254. 10.1086/676809. ISSN 00 21-1753 (2013).

[4] Chepelev NL, Enikanolaiye MI, Chepelev LL, Almohaisen A, Chen Q, Scoggan KA, Coughlan MC, Cao XL, Jin X, Willmore WG (2013). Bisphenol A activates the Nrf1/2-antioxidant response element pathway in HEK 293 cells. Chem. Res. Toxicol..

[5] Ge LC, Chen ZJ, Liu H, Zhang KS, Su Q, Ma XY, Huang HB, Zhao ZD, Wang YY, Giesy JP, Du J, Wang HS (2014). Signaling related with biphasic effects of bisphenol A (BPA) on Sertoli cell proliferation: A comparative proteomic analysis. Biochem. Biophys. Acta..

[6] Kalb AC, Kalb AL, Cardoso TF, Fernandes CG, Corcini CD, Varela Junior AS, Martínez PE (2016). Maternal transfer of bisphenol A during nursing causes sperm impairment in male offspring. Arch. Environ. Contam. Toxicol..

[7] Leem YH, Oh S, Kang HJ, Kim JH, Yoon J, Chang JS (2017). BPA-toxicity via superoxide anion overload and a deficit in beta-catenin signaling in human bone mesenchymal stem cells. Environ. Toxicol..

[8] Gassman, N. R., & Wilson, S. H. Bisphenol A and nongenotoxic drivers of cancer. In *Translational Toxicology and Therapeutics: Windows of Developmental Susceptibility in Reproduction and Cancer*, 415–438 (2017).

[9] Moriyama K, Tagami T, Akamizu T, Usui T, Saijo M, Kanamoto N, Hataya Y, Shimatsu A, Kuzuya H, Nakao K (2002). Thyroid hormone action is disrupted by bisphenol A as an antagonist. J. Clin. Endocrinol. Metab..

[10] Sangai NP, Verma RJ, Trivedi MH (2014). Testing the efficacy of quercetin in mitigating bisphenol A toxicity in liver and kidney of mice. Toxicol. Ind. Health.

[11] Morgan AM, El-Ballal SS, El-Bialy BE, El-Borai NB (2014). Studies on the potential protective effect of cinnamon against bisphenol A- and octylphenol-induced oxidative stress in male albino rats. Toxicol. Rep..

[12] Elobeid M, Hassan Z (2015). Bisphenol A induced oxidative stress and apoptosis in kidney of male rats. J. Environ. Biol..

[13] Asahi J, Kamo H, Baba R, Doi Y, Yamashita A, Murakami D, Hanada A, Hirano T (2010). Bisphenol A induces endoplasmic reticulum stress-associated apoptosis in mouse non-parenchymal hepatocytes. Life Sci..

[14] Salem H, Mohamed A, Saleh E, Shalaby K (2012). Influence of Hesperidin combined with Sinemet on genetical and biochemical abnormalities in rats suffering from Parkinson’s disease. Life Sci. J..

[15] Eslami AC, Pasanphan W, Wagner BA, Buettner GR (2010). Free radicals produced by the oxidation of gallic acid: An electron paramagnetic resonance study. Chem. Cent. J..

[16] Okuda T, Yoshida T, Hatano T (1995). Hydrolyzable tannins and related polyphenols. Fortschritte Der Chemie Organischer Naturstoffe = Progress in the Chemistry of Organic Natural Products. Progres dans la Chimie des Substances Organiques Naturelles.

[17] Inoue M, Sakaguchi N, Isuzugawa K, Tani H, Ogihara Y (2000). Role of reactive oxygen species in gallic acid-induced apoptosis. Biol. Pharm. Bull..

[18] Canbek M, Ustüner MC, Kabay S, Uysal O, Ozden H, Bayramoğlu G, Senturk H, Ozbayar C, Bayramoglu A, Ustuner D, Degirmenci I (2011). The effect of gallic acid on kidney and liver after experimental renal ischemia/reperfusion injury in the rats. Afr. J. Pharm. Pharmacol.

[19] Pal, S., Sarkar, K., Nath, P. P., Mondal, M., Khatun, A., & Paul, G. Bisphenol S impairs blood functions and induces cardiovascular risks in rats. *Toxicol. Rep*. *eCollection* (2017). 10.1016/j.toxrep.2017.10.006 PMC5671619 29152460

[20] El-Yamani M (2011). Cinnamon, cardamom and ginger impacts as evaluated on hyperglycemic rats. Res. Specif. Educ. Mansoura Univ..

[21] Dutta M, Paul G (2019). Gallic acid protects rat liver mitochondria *ex vivo* from bisphenol A induced oxidative stress mediated damages. Toxicol. Rep..

[22] Manucha W, Carrizo L, Ruete C, Molina H, Vallés P (2005). Angiotensin II type I antagonist on oxidative stress and heat shock protein 70 (HSP 70) expression in obstructive nephropathy. Cell Mol. Biol. (Noisy-le-grand).

[23] Phaniendra A, Jestadi DB, Periyasamy L (2015). Free radicals: Properties, sources, targets, and their implication in various diseases. Indian J. Clin. Biochem..

[24] Ola-Davies OE, Olukole SG (2018). Gallic acid protects against bisphenol A-induced alterations in the cardio-renal system of Wistar rats through the antioxidant defense mechanism. Biomed. Pharmacother..

[25] Yıldırım Ö (2003). Changes in nitric oxide level of different tissues in diabetic rats. Biotechnol. Biotechnol. Equip..

[26] Szentiványi M, Park F, Maeda CY, Cowley AW (2000). Nitric oxide in the renal medulla protects from vasopressin-induced hypertension. Hypertension.

[27] Rubbo H, Radi R, Trujillo M, Telleri R, Kalyanaraman B, Barnes S, Kirk M, Freeman BA (1994). Nitric oxide regulation of superoxide and peroxynitrite-dependent lipid peroxidation. Formation of novel nitrogen-containing oxidized lipid derivatives. J. Biol. Chem..

[28] De Oliveira LM, De Oliveira TS, Da Costa RM, Gil E, Costa EA, Rde C, Filgueira FP, Ghedini PC (2016). The vasorelaxant effect of gallic acid involves endothelium-dependent and -independent mechanisms. Vascul. Pharmacol..

[29] Peerapanyasut W, Kobroob A, Palee S, Chattipakorn N, Wongmekiat O (2019). Activation of sirtuin 3 and maintenance of mitochondrial integrity by N-acetylcysteine protects against bisphenol A-induced kidney and liver toxicity in rats. Int. J. Mol. Sci..

[30] Khan S, Beigh S, Chaudhari BP, Sharma S, Aliul Hasan Abdi S, Ahmad S, Ahmad F, Parvez S, Raisuddin S (2016). Mitochondrial dysfunction induced by Bisphenol A is a factor of its hepatotoxicity in rats. Environ. Toxicol..

[31] Maurya, H., Mangal, V., Gandhi, S., Prabhu, K., & Ponnudurai, K. Prophylactic Antioxidant Potential of gallic acid in Murine Model of Sepsis. *Int. J. Inflamm.* article ID 580320. 10.1155/2014/580320 (2014). 10.1155/2014/580320 PMC4074961 25018890

[32] Ajibade TO, Oyagbemi AA, Omobowale TO, Asenuga ER, Afolabi JM, Adedapo AA (2016). Mitigation of diazinon-induced cardiovascular and renal dysfunction by gallic acid. Interdiscip. Toxicol..

[33] Sheweita SA, Almasmari AA, El-Banna SG (2018). Tramadol-induced hepato- and nephrotoxicity in rats: Role of curcumin and gallic acid as antioxidants. PLoS ONE.

[34] Moon MK, Kim MJ, Jung IK, Koo YD, Ann HY, Lee KJ, Kim SH, Yoon YC, Cho BJ, Park KS, Jang HC, Park YJ (2012). Bisphenol A impairs mitochondrial function in the liver at doses below the no observed adverse effect level. J. Korean Med. Sci..

[35] Stenvinkel P, Ketteler M, Johnson RJ, Lindholm B, Pecoits-Filho R, Riella M, Hiemburger O, Cederholm T, Grindt M (2005). IL-10, IL-6, and TNF-alpha: Central factors in the altered cytokine network of uremia—The good, the bad, and the ugly. Kid.. Int. J..

[36] Rashid S, Idris-Khodja N, Auger C, Kevers C, Pincemail J, Alhosin M, Boehm N, Oswald-Mammosser M, Schini-Kerth VB (2018). Polyphenol-rich blackcurrant juice prevents endothelial dysfunction in the mesenteric artery of cirrhotic rats with portal hypertension: Role of oxidative stress and the angiotensin system. J. Med. Food.

[37] Wahbby MM, Abdallah ZM, Abdou HM, Yousef MI, Newairy AA (2017). Mitigating potential of Ginkgo biloba extract and melatonin against hepatic and nephrotoxicity induced by bisphenol A in male rats. Egypt. J. Basic Appl. Sci..

[38] Ola-Davies OE, Olukole SG, Lanipekun DO (2018). Gallic Acid Ameliorates bisphenol A-induced toxicity in Wistar rats. Iran. J. Toxicol..

[39] Ahmed WM, Moselhy WA, Nabil TM (2015). Bisphenol A toxicity in adult male rats: Hematological, biochemical and histopathological approach. Glob. Vet..

[40] O’Morchoe PJ, Thursh DR, Levy AH (1994). The Urbana Atlas of Pathology.

[41] Sangai NP, Verma RJ (2012). Protective effect of quercetin on bisphenol A-caused alterations in succinate dehydrogenase and adenosine triphosphatase activities in liver and kidney of mice. Acta Pol. Pharm..

[42] Shimizu S, Eguchi Y, Kamiike W, Waguri S, Uchiyama Y, Matsuda H, Tsujimoto Y (1996). Retardation of chemical hypoxia induced necrotic cell death by Bcl-2 and ICE inhibitors: Possible involvement of common mediators in apoptotic and necrotic signal transductions. Oncogene.

[43] Dogrul BN, Kiliccalan I, Asci ES (2016). The protective effect of gallic acid against endocrine disrupting damages of bisphenol A and possible mechanism. J. Contemp. Med. Educ..

[44] Gu R, Zhang M, Meng H, Xu D, Xie Y (2018). Gallic acid targets acute myeloid leukemia via Akt/mTOR-dependent mitochondrial respiration inhibition. Biomed. Pharmacother..

[45] Korkmaz A, Aydoğan M, Kolankaya D, Barlas N (2011). Vitamin C coadministration augments bisphenol A, nonylphenol, and octylphenol induced oxidative damage on kidney of rats. Environ. Toxicol..

[46] Hassan ZK, Elobeid MA, Virk P, Omer SA, ElAmin M, Daghestani MH, AlOlayan EM (2012). Bisphenol A induces hepatotoxicity through oxidative stress in rat model. Oxid. Med. Cell. Longev..

[47] Wetherill YB, Akingbemi BT, Kanno J, McLachlan JA, Nadal A, Sonnenschein C, Watson CS, Zoeller RT, Belcher SM (2007). *In vitro* molecular mechanisms of bisphenol A action. Reprod. Toxicol..

[48] Colletti LM, Kunkel SL, Walz A, Burdick MD, Kunkel RG, Wilke CA, Strieter RM (1996). The role of cytokine networks in the local liver injury following hepatic ischemia/reperfusion in the rat. Hepatology.

[49] Jaeschke H, Smith CW (1997). Mechanisms of neutrophil-induced parenchymal cell injury. J. Leukoc. Biol..

[50] Asci H, Ozmen O, Ellidag HY, Aydin B, Bas E, Yilmaz N (2017). The impact of gallic acid on the methotrexate-induced kidney damage in rats. J. Food Drug Anal..

[51] Kattaia AAA, Abdel Baset SA (2014). Effect of bisphenol A on the lung of adult male albino rats and the possible protective role of geraniol: A histological and immunohistochemical study. Egypt. J. Histol..

[52] Ramos JG, Varayoud J, Sonnenschein C, Soto AM, Muñoz De Toro M, Luque EH (2001). Prenatal exposure to low doses of bisphenol A alters the periductal stroma and glandular cell function in the rat ventral prostate. Biol. Reprod..

[53] Selman M, King TE, Pardo A (2001). Idiopathic pulmonary fibrosis: Prevailing and evolving hypotheses about its pathogenesis and implications for therapy. Ann. Intern. Med..

[54] Fadda WA, Essawy AS, Faried MA (2019). Green tea extract protects the renal cortex against bisphenol A-induced nephrotoxicity in the adult male albino rat: A histological and immunohistochemical study. Eur. J. Anat..

[55] Palmer RM, Ferrige AG, Moncada S (1987). Nitric oxide release accounts for the biological activity of endothelium-derived relaxing factor. Nature.

[56] Negre-Salvayre A, Coatrieux C, Ingueneau C, Salvayre R (2008). Advanced lipid peroxidation end products in oxidative damage to proteins. Potential role in diseases and therapeutic prospects for the inhibitors. Br. J. Pharmacol..

[57] El-Banhawy MA, Ganzuri MA (1980). Pathological effects of insecticides on acid phosphatase containing particles in mammalian liver cells. Proc. Egypt. Soc. Environ. Sci..

[58] Peerapanyasut W, Kobroob A, Palee S, Chattipakorn N, Wongmekiat O (2019). N-acetylcysteine attenuates the increasing severity of distant organ liver dysfunction after acute kidney injury in rats exposed to bisphenol A. Antioxidants.

[59] Bosch-Panadero E, Mas S, Civantos E, Abaigar P, Camarero V, Ruiz-Priego A, Ortiz A, Egido J, González-Parra E (2018). Bisphenol A is an exogenous toxin that promotes mitochondrial injury and death in tubular cells. Environ. Toxicol..

[60] Olea-Herrero N, Arenas MI, Muñóz-Moreno C, Moreno-Gómez-Toledano R, González-Santander M, Arribas I, Bosch RJ (2014). Bisphenol-A induces podocytopathy with proteinuria in mice. J. Cell. Physiol..

[61] Kourouma, A., Quan, C., Duan, P., Qi, S., Yu, T., Wang, Y., & Yang, K. Bisphenol A induces apoptosis in liver cells through induction of ROS. *Adv. Toxicol.* Article ID 901983 (2015). 10.1155/2015/901983

[62] Mard, S. A., Mojadami, S., Farbood, Y. & Naseri, M. K. G. The anti-inflammatory and anti-apoptotic effects of gallic acid against mucosal inflammation-and erosions-induced by gastric ischemia-reperfusion in rats. In *Veterinary Research Forum*, Vol. 6, No. 4, 305–311 (Faculty of Veterinary Medicine, Urmia University, 2015). PMC4769336 26973766

[63] Erol-Dayi Ö, Arda N, Erdem G (2012). Protective effects of olive oil phenolics and gallic acid on hydrogen peroxide-induced apoptosis. Eur. J. Nutr..

[64] Mahmoudi A, Ghorbel H, Bouallegui Z (2015). Oleuropein and hydroxytyrosol protect from bisphenol a effects in livers and kidneys of lactating mother rats and their pups. Exp. Toxicol. Pathol..

[65] Karimi-Khouzani O, Heidarian E, Amini SA (2017). Anti-inflammatory and ameliorative effects of gallic acid on fluoxetine-induced oxidative stress and liver damage in rats. Pharmacol. Rep..

[66] Parasuraman S, Raveendran R, Kesavan R (2010). Blood sample collection in small laboratory animals. J. Pharmacol. Pharmacother..

[67] Zaghlool SS, Shehata BA, Abo-Seif AA, Abd El-Latif HA (2015). Protective effects of ginger and marshmallow extracts on indomethacin-induced peptic ulcer in rats. J. Nat. Sci. Biol. Med..

[68] Tietz NW (1986). Textbook of Clinical Chemistry.

[69] Tietz NW (1990). Clinical Guide to Laboratory Tests.

[70] Kalyanaraman B, Darley-Usmar V, Davies KJA, Dennery PA, Forman HJ, Grisham MB, Mann GE, Moore KL, Roberts LJ, Ischiropoulos H (2012). Measuring reactive oxygen and nitrogen species with fluorescent probes: Challenges and limitations. Free Radic. Biol. Med..

[71] Ding AH, Nathan CF, Stuehr DJ (1988). Release of reactive nitrogen intermediates and reactive oxygen intermediates from mouse peritoneal macrophages. Comparison of activating cytokines and evidence for independent production. J. Immunol..

[72] Ohkawa H, Ohishi N, Yagi K (1979). Assay for lipid peroxides in animal tissues by thiobarbituric acid reaction. Anal. Biochem..

[73] Stadtman ER, Levine RL (2000). Protien oxidation. Ann. N. Y. Acad. Sci..

[74] Beers RF, Sizer IW (1952). Aspectrophotometric method for measuring the breakdown of hydrogen peroxide by catalase. J. Biol. Chem..

[75] Misra HP, Fridovich I (1972). The role of superoxide anion in the autoxidation of epinephrine and a simple assay for superoxide dismutase. J. Biol. Chem..

[76] Ellman GL (1959). Tissue sulfhydryl groups. Arch. Biochem. Biophys..

[77] Lowry OH, Rosenbrough NG, Farr AL, Randall RJ (1951). Protein measurement with Folin phenol reagent. J. Biol..

[78] Abd-Elhafeez HH, Soliman SA (2017). New description of telocyte sheaths in the bovine uterine tube: An immunohistochemical and scanning microscopic study. Cells Tissues Organs.

[79] Drury RA, Willington EA, Carleton S (1980). Histological Techniques.

[80] Gabe M (1976). Histological Techniques.

[81] McManus JFA (1946). Histological demonstration of mucin after periodic acid. Nature.

[82] Jansson A, Sun XF (2002). Bax expression decreases significantly from primary tumor to metastasis in colorectal cancer. J. Clin. Oncol..

[83] Krajewski S, Krajewska M, Shabaik A, Miyashita T, Wang HG, Reed JC (1994). Immunohistochemical determination of *in vivo* distribution of bax, a dominant inhibitor of bcl-2. Am. J. Pathol..

[84] Gupta PD (1983). Ultrastructural study on semithin section. Sci. Tools.

